# Physiological Action of Sulphate of Quinine

**Published:** 1880-03

**Authors:** 


### PHYSIOLOGICAL ACTION OF SULPHATE OF QUININE.

*Progrès Médical.*

1. *Skin.*--Applied to the dermis, deprived of its epithelium, the sulphate of quinine provokes lively pains and an eschar of ordinary depth, as has been shown by the experiments of M. Trousseau. It has been pretended that sulphate of quinine, administered topically in form of lotions, pomades, ointments, etc., was absorbed, and produced therapeutic effects. Such is, in reality, the case to a small degree with children, but not to an appreciable extent with adults.

2. *Digesteric Apparatus.*—On the digestive mucus the sulphate of quinine acts as an irritant. Vomiting may sometimes follow its administration, but, according to many authors, it is diarrhœa that is most observed. Bretonneau insists upon this purgative action in persons inhabiting marshy districts, who, as he thinks, annihilate the therapeutic action of this salt and necessitate the adjunct of opium to establish its tolerance. The author had never seen the sulphate of quinine produce diarrhœa; had rather seen it constipate. But the vomiting it may occasion, the gastralgia that it may determine, justify the employment of opium and demand that it should be taken at the beginning of or during the course of meals.

3. *Circulation Calorification.*—Sulphate of quinine produces a sedative effect upon the cardio-vascular and calorific apparatus, which manifests itself by a slackening in the heart's action, a contraction of the smaller blood vessels and a lowering of the temperature. Also, on these conditions it is excellent as an antipyretic and decongestive of the nervous centres and the ocular globe. Sometimes this capillary anæmia and refrigeration are followed by an access of heat and marked fever. M. Briquet considers the sulphate of quinine simply a sedative of the nervous system and notably of the ganglionary portion, which controls the functions of circulation, nutrition and calorification. He denies that sulphate of quinine has any tonic effect.

4. *Urinary Organs.*—Sulphate of quinine is eliminated in the urine and can be easily found by different tests. Its passage may be accompanied by a certain sensibility of the urinary tracts. The urinary liquid is augmented and the proportion of uric acid diminished. Sulphate of quinine is also eliminated, though in smaller quantities, by sweat, milk, saliva and tears. Traces of sulphate of quinine do not appear in the urine when it is applied to the skin by friction, unless such friction be continued for a long time, and then the traces are quite faint.

5. *Nervous System.*—Experiments on animals and numerous clinical observations have induced M. Briquet to claim for sulphate of quinine an action rapid and energetic upon the nervous system (cerebro-spinal) and the grand sympathetic. Sulphate of quinine excites the nervous system, produces capillary anæmia, then, the dose spent, a paralysis of those same vessels succeeds. In augmenting the dose progressively the picture of quinia intoxication presents itself to view in all its different phases, from the most insignificant phenomena to the most grave accidents. These are at first cephalagia, ringing in the ears, vertigo, slight stumbling, then numbness, deafness, stupor and obscurity of vision; finally, annihilization of all the functions of relation and veritable coma, which may be interrupted by convulsive efforts or delirium simulating real meningitis and ending in death.

*Resumé.*—Quinic action is directed upon the medulla, the cerebro-spinal systems and the grand sympathetic, which it excites; from this source come its anæmic and decongestive properties. The author believes, however, that the troubles of the brain have been wrongly attributed to the only modifications in the circulation of that organ, cerebral anæmia. Besides that action, it exercises a direct one, and one of its own, upon the elements of the nervous system. As regards toxical agents in general, and, of course, quinine belongs in that category, bear in mind that their direct action upon the nervous centres is of more importance than their action upon the vascular tension.

How, indeed, explain the intellectual and moral perturbations, so different in their physiognomy, even taking into consideration certain primordial common facts that follow the ingestion of belladonna, opium, cannabis indica, alcohol, etc., if the entire action of these substances is reduced to augmentations or diminuitions of tension in the cerebral vessels? How understand that alcohol and opium, both causing cerebral congestion, the best antidote of alcohol is opium? The separate action on the nervous centres answers these points conclusively.

BOYLAND.

